# Functional neuroanatomy of auditory scene analysis in Alzheimer's disease

**DOI:** 10.1016/j.nicl.2015.02.019

**Published:** 2015-02-28

**Authors:** Hannah L. Golden, Jennifer L. Agustus, Johanna C. Goll, Laura E. Downey, Catherine J. Mummery, Jonathan M. Schott, Sebastian J. Crutch, Jason D. Warren

**Affiliations:** a Dementia Research Centre, UCL Institute of Neurology, University College London, London, UK

**Keywords:** Auditory scene analysis, Cocktail party effect, Alzheimer's disease, fMRI

## Abstract

Auditory scene analysis is a demanding computational process that is performed automatically and efficiently by the healthy brain but vulnerable to the neurodegenerative pathology of Alzheimer's disease. Here we assessed the functional neuroanatomy of auditory scene analysis in Alzheimer's disease using the well-known ‘cocktail party effect’ as a model paradigm whereby stored templates for auditory objects (e.g., hearing one's spoken name) are used to segregate auditory ‘foreground’ and ‘background’. Patients with typical amnestic Alzheimer's disease (*n* = 13) and age-matched healthy individuals (*n* = 17) underwent functional 3T-MRI using a sparse acquisition protocol with passive listening to auditory stimulus conditions comprising the participant's own name interleaved with or superimposed on multi-talker babble, and spectrally rotated (unrecognisable) analogues of these conditions. Name identification (conditions containing the participant's own name contrasted with spectrally rotated analogues) produced extensive bilateral activation involving superior temporal cortex in both the AD and healthy control groups, with no significant differences between groups. Auditory object segregation (conditions with interleaved name sounds contrasted with superimposed name sounds) produced activation of right posterior superior temporal cortex in both groups, again with no differences between groups. However, the cocktail party effect (interaction of own name identification with auditory object segregation processing) produced activation of right supramarginal gyrus in the AD group that was significantly enhanced compared with the healthy control group. The findings delineate an altered functional neuroanatomical profile of auditory scene analysis in Alzheimer's disease that may constitute a novel computational signature of this neurodegenerative pathology.

## Highlights

• Auditory scene analysis is affected in Alzheimer's disease (AD) but little studied.
• We addressed this using fMRI of the ‘cocktail party effect’ in AD vs older controls.
• Relative to controls, AD patients showed enhanced activation of supramarginal gyrus.
• Auditory scene analysis is a new, informative probe of brain network function in AD.

## 1 Introduction

Decoding the auditory world poses a formidable problem of neural computation. Our brains normally solve this problem efficiently and automatically but the neural basis of ‘auditory scene analysis’ remains incompletely understood. The disambiguation of sound sources within the complex mixture that generally arrives at our ears is an essential prerequisite for identification of those sources and a fundamental task of auditory scene analysis (Bregman, 1994). One of the best known instances of this process in action is the so-called ‘cocktail party effect’ whereby our own name spoken across a noisy room captures attention and may even lead to successful tracking of the relevant conversation against the surrounding babble (Cherry, 1953; Moray, 1959). The cocktail party effect is a celebrated example of a much wider category of auditory phenomena that depend on generic computational processes that together segregate an acoustic target or ‘foreground’ sound from the acoustic ‘background’: these processes are likely to include representation of spectral and temporal regularities in the sound mixture and matching to previously stored auditory ‘templates’ (for example, specific speech or vocal sounds) prior to engagement of attentional resources (Billig et al., 2013; Griffiths and Warren, 2002; Kumar et al., 2007). Functional neuroimaging studies to define neuroanatomical substrates of auditory scene analysis in the healthy brain have implicated a distributed, dorsally directed cortical network including planum temporale and posterior superior temporal gyrus, supramarginal gyrus, intraparietal sulcus and prefrontal projection targets (Dykstra et al., 2011; Gutschalk et al., 2007; Hill and Miller, 2010; Kondo and Kashino, 2009; Kong et al., 2014; Linden et al., 1999; Overath et al., 2010; Wilson et al., 2007; Wong et al., 2009). While frontal cortex is thought to drive top-down attentional processes (Hill and Miller, 2010; Obleser et al., 2007; Schönwiesner et al., 2007), the precise role of parietal cortex in auditory scene analysis is more contentious and might include primary labelling of salient events (Cohen, 2009; Downar et al., 2000), integration of signal representations for programming behavioural responses (Cusack, 2005; Lee et al., 2014) or attentional modulation (Hill and Miller, 2010; Nakai et al., 2005). With particular reference to the cocktail party effect, speech intelligibility has been shown to engage more ventral and anterior superior temporal cortex in the dominant hemisphere (Scott et al., 2000), but is influenced by the nature of the background masker (speech versus non-speech: Scott and McGettigan, 2013; Scott et al., 2009). Lexical processes may modulate auditory scene analysis, perhaps via template matching algorithms (Billig et al., 2013; Griffiths and Warren, 2002) as well as additional parietal and prefrontal mechanisms engaging in speech in noise processing, particularly under conditions of increased attentional demand (Binder et al., 2004; Davis et al., 2011; Nakai et al., 2005; Scott et al., 2004; Scott and McGettigan, 2013).

On behavioural as well as neuroanatomical grounds, the computational processing required for auditory scene analysis is likely to be particularly vulnerable to the neurodegenerative disease process in Alzheimer's disease (AD). Patients with AD commonly experience difficulties in following conversations under degraded listening conditions such as a busy room or noisy telephone line. Both generic deficits of central auditory processing and specific deficits of auditory scene analysis have been demonstrated in AD (Gates et al., 1996, 2008, 2011; Golden et al., 2015; Goll et al., 2011, 2012; Golob et al., 2007, 2009; Kurylo et al., 1993; Strouse et al., 1995); these develop early in the course of disease and are likely to interact with impairments of attention and working memory (Conway et al., 2001; Goll et al., 2012; Stopford et al., 2012). Deficits of auditory scene analysis are in accord with the neuroanatomy of AD, which blights a large-scale, functionally coherent brain network linking mesial temporal lobe structures with retrosplenial, temporo-parietal and medial prefrontal cortices (Buckner et al., 2008; Greicius and Menon, 2004; Raichle et al., 2001; Seeley et al., 2009). Regional deposition of pathogenic proteins, hypometabolism and atrophy within this network in AD closely overlaps regions implicated in auditory scene analysis and speech-in-noise processing in the healthy brain, and involvement of temporo-parietal cortical junction zones is likely to be particularly pertinent (Herholz et al., 2002; Scahill et al., 2002; Warren et al., 2012). Indeed, modulation of activity in these areas has been linked to the efficiency of speech-in-noise processing even in apparently healthy older individuals (Wong et al., 2009). However, the pathophysiology of this culprit brain network in AD remains to be worked out in detail. While involvement of this network is relatively selective in AD, it is unlikely that the network behaves as an amorphous unit (Warren et al., 2012); moreover its core function or functions have not been defined. Although it has been designated the ‘default mode network’, showing correlated activity in the healthy ‘resting’ brain and deactivation with certain tasks (Buckner et al., 2008; Raichle et al., 2001; Shulman et al., 1997), this network has also been implicated in various ‘active’ processes including maintenance of internal sensory representations (Buckner et al., 2008; Buckner and Carroll, 2007; Spreng and Grady, 2010; Zvyagintsev et al., 2013) and more specifically in aspects of auditory scene analysis, both in the healthy brain (Salvi et al., 2002; Wong et al., 2009; Zündorf et al., 2013) and in patients with AD (Goll et al., 2012).

Here we used the cocktail party effect to delineate the functional neuroanatomy of auditory scene analysis in a cohort of patients with AD in relation to healthy older individuals. Previous work in AD has addressed psychophysical deficits of auditory scene analysis using relatively simple paradigms and structural neuroanatomical correlation (Gates et al., 2008, 2011; Goll et al., 2012). In this study we set out to use a realistic auditory scene analysis paradigm in the context of fMRI, in order to probe functional brain mechanisms directly. This paradigm was motivated by a cognitive model of the cocktail party effect according to which stored templates for auditory objects (e.g., spoken words) are used to disambiguate those objects from other sounds in the environment during parsing of the auditory scene (segregation of auditory ‘foreground’ and ‘background’: Griffiths and Warren, 2002). We used participant's own names as salient acoustic targets (Moray, 1959; Wood and Cowan, 1995) against naturalistic multi-talker babble; a sparse fMRI acquisition protocol to minimise confounding effects engendered by streaming auditory stimuli against scanner noise (Hall et al., 1999); and a passive-listening design to minimise any confounding effects from output task in these cognitively impaired patients. Based on previous neuroanatomical work in the healthy brain and in AD, we hypothesised that patients with AD and healthy older individuals would show similar profiles of auditory cortex activation by sound and representation of name identity per se; but that AD would have a distinct pathophysiological signature during auditory scene analysis, in temporo-parietal cortical regions separable from more anterior superior temporal cortex engaged by name identity coding (Dykstra et al., 2011; Goll et al., 2012; Overath et al., 2010; Scott et al., 2000, 2009; Wong et al., 2009). In particular, we hypothesised that AD would produce an altered interaction of auditory name template matching with object segregation underpinning the cocktail party effect.

## 2 Methods

### 2.1 Participants

Thirteen consecutive patients (mean (standard deviation) age 66 (5.8) years; five female) fulfilling consensus clinical criteria for early to moderately severe, typical Alzheimer's disease (AD) led by predominant episodic memory loss with additional cognitive dysfunction (Dubois et al., 2007) and 17 age-matched healthy individuals (68 (3.9) years; seven female) with no history of neurological or psychiatric illness participated in the study. All participants were right-handed and no participant had a clinical history of peripheral hearing loss; none was a professional musician. Detailed general neuropsychological assessment in the AD group corroborated the clinical diagnosis in all cases; demographic, clinical and neuropsychological details for the experimental groups are summarised in Table 1. At the time of participation, 12 patients were receiving symptomatic treatment with an acetylcholinesterase inhibitor (one was also receiving memantine). CSF examination was undertaken in six patients with AD and revealed a total tau: beta-amyloid ratio >1 (compatible with underlying AD pathology) in all cases. All participants gave informed consent in accordance with the Declaration of Helsinki.

### 2.2 Assessment of peripheral hearing

All participants had pure-tone audiometry using a procedure adapted from a commercial screening audiometry software package (AUDIO-CDTM®, http://www.digital-recordings.com/audiocd/audio.html). The test was administered via headphones from a notebook computer in a quiet room. Five frequency levels (500, 1000, 2000, 3000, 4000 Hz) were assessed: at each frequency, participants were presented with a continuous tone that slowly and linearly increased in intensity. Participants were instructed to indicate as soon as they were sure they could detect the tone; this response time was measured and stored for offline analysis. Hearing was assessed in the right ear in each participant.

### 2.3 Experimental design and stimuli

In designing the experimental paradigm we manipulated two key components of the cocktail party effect: separation of a particular ‘foreground’ auditory object (a spoken word) from a complex sound mixture or acoustic ‘background’; and matching of foreground object (own name) identity with a previously stored ‘template’. In order to isolate the neural processes involved in these computations, we created two closely matched auditory baseline conditions: by presenting ‘foreground’ sounds interleaved with (rather than superimposed on) the acoustic background; and by spectral rotation of participants' spoken names to generate acoustically similar but unfamiliar (and unintelligible) sound objects. Under this design, the cocktail party effect (detection of own name in a busy auditory scene) represents the interaction of processes that mediate auditory object segregation and template matching.

Stimuli were created as digital wave files and edited in MATLAB7.0® (http://www.mathworks.co.uk); examples of stimuli are available in Supplementary Material on-line. Each participant's own first name was recorded in a sound-proof room, by the same young adult female speaker using a Standard Southern English accent. Recorded name sounds were spectrally rotated using a previously described procedure that preserves spectral and temporal complexity but renders speech content unintelligible (Blesser, 1972). An acoustic ‘background’ of speech babble was created by superimposing recordings of 16 different female speakers reading passages of English from the EUROM database of English speech (Chan et al., 1995) using a previously described method (Rosen et al., 2013); no words were intelligible from the sound mixture. Babble samples were spectrally rotated in order to provide an acoustic background for the spectrally rotated name sounds that reduced any spectral ‘pop-out’ effects. The signal-to-noise ratio of names to background babble was fixed at 17 dB, corresponding to a moderately noisy (e.g., cocktail party) environment (International Telecommunication Union, 1986).

**Fig. 1:**
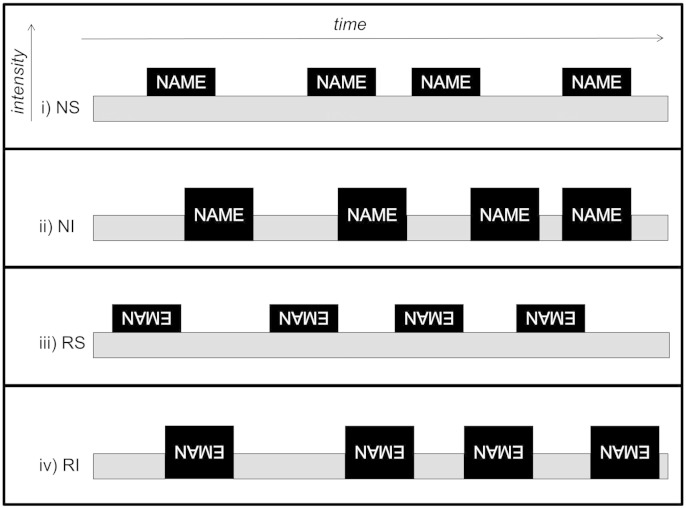
Schematic representation of fMRI stimulus conditions, showing representative trials. Dark grey boxes signify presentations of participant's own name, in either natural or spectrally rotated (inverted) form; light grey boxes represent the acoustic background (multi-talker babble). Onsets of name exemplars were varied randomly between trials; each trial was 8 s in total duration. NS, own natural name sounds superimposed on babble; NI, own natural name sounds interleaved with babble; RS, spectrally rotated name sounds superimposed on babble; RI, spectrally rotated name sounds interleaved with babble.

To create experimental trials, name and spectrally rotated name sounds were added to corresponding (raw or spectrally rotated) babble samples by either superimposing on or interleaving with babble; name sounds were repeated four times within a single trial and the total duration of each trial was fixed at 8 s (duration of individual name exemplars 0.6–0.9 s; experimental trials schematised in Fig. 1). Concatenated sound samples were windowed with 20 ms onset–offset temporal ramps to prevent click artefacts, and all wave files were digitally sampled at 44,100 Hz with fixed mean (root-mean-square) intensity over all trials. These procedures yielded four experimental conditions in a factorial relation: own natural name superimposed on babble, NS; own natural name interleaved with babble, NI; spectrally rotated name superimposed on (spectrally rotated) babble, RS; spectrally rotated name interleaved with (spectrally rotated) babble, RI. Twenty unique trials were created for each condition, by randomly varying the onsets of the four name sounds within the 8 s trial interval. An additional rest baseline condition comprising 8 s silent intervals was also included.

### 2.4 Experimental procedures

#### 2.4.1 Stimulus presentation

In the fMRI session, experimental trials were presented from a notebook computer running the Cogent v1.25 extension of MATLAB (Vision Lab, University College London, UK), each triggered by the MR scanner on completion of the previous image acquisition in a ‘sparse’ acquisition protocol. Sounds were delivered binaurally via electrodynamic headphones (http://www.mr-confon.de) at a comfortable listening level (at least 70 dB) that was fixed for all participants; two identical scanning runs were administered, each comprising 20 trials for each sound condition plus 10 silence trials, yielding a total of 180 trials for the experiment. Participants were instructed to listen to the sound stimuli with their eyes open; there was no in-scanner output task and no behavioural responses were collected.

#### 2.4.2 Brain image acquisition

Brain images were acquired on a 3 Tesla TIM Trio MRI scanner (Siemens Healthcare, Erlangen, Germany) using a 12-channel RF receive head coil. For each of the two functional runs, 92 single-shot gradient-echo planar image (EPI) volumes were acquired each with 48 oblique transverse slices covering the whole brain (slice thickness 2 mm, inter-slice gap 1 and 3 mm in-plane resolution, TR/TE 70/30 ms, echo spacing 0.5 ms, matrix size 64 × 64 pixels, FoV 192 × 192 mm, phase encoding (PE) direction anterior–posterior). A slice tilt of −30° (*T* > *C*), z-shim gradient moment of +0.6 mT/m ms and positive PE gradient polarity were used to minimise susceptibility-related loss of signal and blood-oxygen-level-dependent (BOLD) functional sensitivity in the temporal lobes, following optimisation procedures described previously (Weiskopf et al., 2006). Sparse-sampling EPI acquisition with repetition time 11.36 s (corresponding to an inter-scan gap of 8 s) was used to reduce any interaction between scanner acoustic noise and auditory stimulus presentations. The initial two brain volumes in each run were performed to allow equilibrium of longitudinal T1 magnetisation but discarded from further analysis. A B0 field-map was acquired using a gradient double-echo FLASH sequence (TE1 = 10 ms, TE2 = 12.46 ms, 3 × 3 × 2 mm resolution, 1 mm gap; matrix size = 64 × 64 pixels; FoV = 192 × 192 mm) to allow post-processing geometric distortion corrections of EPI data due to B0 field inhomogeneities.

Volumetric brain MR images were also obtained in each participant to allow coregistration of structural with functional neuroanatomical data. The structural acquisition was based on a multi-parameter mapping protocol (Weiskopf et al., 2011; Weiskopf and Helms, 2008), including a 3D multi-echo FLASH sequence with predominant T1 (TR 18.7 ms, flip angle 20°) weighting, six alternating gradient echoes at equidistant echo times and 1 mm isotropic voxels.

#### 2.4.3 Behavioural assessment

Following the scanning session, each participant's ability to perceive and discriminate the experimental conditions presented during scanning was assessed using a two alternative forced choice psychoacoustic procedure. Twenty auditory stimuli representing all sound conditions (five NS, five NI, five RS, five RI) were derived from trials presented in the scanner and administered in randomised order in two short tests. In the first test, the task (name detection) was to determine whether or not the participant's own name was present (discrimination of NS/NI from RS/RI conditions); in the second test, the task (segregation detection) was to determine whether the two kinds of sounds (name and babble) were superimposed or interleaved (‘Are the sounds over the top or in-between?’; discrimination of NS/RS from NI/RI conditions), assisted by a visual guide (see Fig. S1 in Supplementary Material on-line). It was established that all participants understood the tasks prior to commencing the tests; during the tests, no feedback about performance was given and no time limits were imposed. Participant responses were recorded for off-line analysis.

**Fig. S1:**
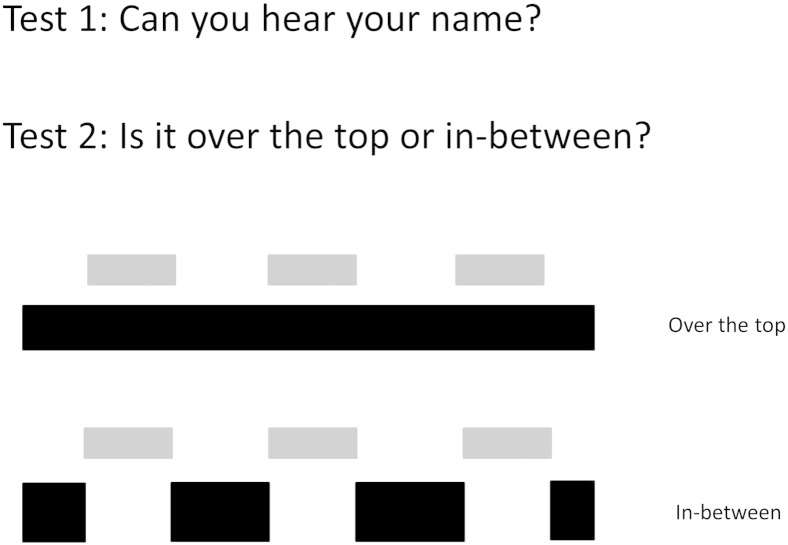
Visual guide shown to participants in post-scan behavioural testing to assess auditory segregation detection (discrimination between superimposed and interleaved sound conditions). The ‘foreground’ sound (grey) was either the participant's natural spoken name or its spectrally rotated (unintelligible) analogue; the ‘background’ sound (black) was either 16-talker babble or its spectrally rotated analogue. In this test, the task instruction on each trial was to decide whether the two kinds of sounds (‘grey’ and ‘black’) were ‘over the top’ (superimposed) or ‘in-between’ (interleaved).

Inline Supplementary Figure S1

### 2.5 Data analyses

#### 2.5.1 fMRI data analysis

Brain imaging data were analysed using statistical parametric mapping software (SPM8; http://www.fil.ion.ucl.ac.uk/spm). In initial image pre-processing, the EPI functional series for each participant was realigned using the first image as a reference and images were unwarped incorporating field-map distortion information (Hutton et al., 2002). The DARTEL toolbox (Ashburner, 2007) was used to spatially normalise all individual functional images to a group mean template image in Montreal Neurological Institute (MNI) standard stereotactic space; to construct this group brain template, each individual's T1 weighted MR image was first co-registered to their EPI series and segmented using DARTEL tools (New Segment) and this segment was then used to estimate a group template that was aligned to MNI space. Functional images were smoothed using a 6 mm full-width-at-half-maximum Gaussian smoothing kernel. For the purpose of rendering statistical parametric functional maps, a study-specific mean structural brain image template was created by warping all bias-corrected native space whole-brain images to the final DARTEL template and calculating the average of the warped brain images.

Pre-processed functional images were entered into a first-level design matrix incorporating the five experimental conditions (NS, NI, RS, RI and the baseline silence condition) modelled as separate regressors convolved with the standard haemodynamic response function, and also including six head movement regressors generated from the realignment process. For each participant, first-level *t*-test contrast images were generated for the main effects of auditory stimulation [(NS + NI + RS + RI) − silence], identification of own name [(NS + NI) − (RS + RI)] and segregation of auditory foreground from background [(NS + RS) − (NI + RI)]. In the absence of a specific output task during scanning, we use ‘identification’ here to indicate specific processing of own-name identity in relation to an acoustically similar perceptual baseline. In addition, contrast images were generated for the interaction of identification and segregation processes [(NS − RS) − (NI − RI)]: we argue that this interaction captures the computational process that supports the cocktail party effect proper. Both ‘forward’ and ‘reverse’ contrasts were assessed in each case. Contrast images for each participant were entered into a second-level random-effects analysis in which effects within each experimental group and between the healthy control and AD groups were assessed using voxel-wise *t*-test contrasts.

Contrasts were assessed at peak voxel statistical significance threshold *p* < 0.05 after family-wise error (FWE) correction for multiple voxel-wise comparisons in two anatomical small volumes of interest, specified by our prior hypotheses (Dykstra et al., 2011; Goll et al., 2012; Overath et al., 2010; Scott et al., 2000, 2009; Wong et al., 2009). These regional volumes were created using MRICron® (http://www.mccauslandcenter.sc.edu/mricro/mricron/) and comprised temporo-parietal junction (including superior temporal and adjacent inferior parietal cortex posterior to Heschl's gyrus and supramarginal gyrus; the putative substrate for auditory scene analysis) and superior temporal gyrus anterior and lateral to Heschl's gyrus (the putative substrate for name identity coding). For the purpose of assessing overall auditory stimulation, a combined regional volume with addition of Heschl's gyrus was used for the contrast [(NS + NI + RS + RI) − silence].

#### 2.5.2 Voxel-based morphometry of structural MR images

Structural brain images were compared between the patient and healthy control groups in a voxel-based morphometric (VBM) analysis to obtain an AD-associated regional atrophy map: normalisation, segmentation and modulation of grey and white matter images were performed using default parameter settings in SPM8, with a Gaussian smoothing kernel of 6 mm full-width-at-half-maximum. Groups were compared using voxel-wise two-sample *t*-tests, including covariates of age, gender, and total intracranial volume. Statistical parametric maps of brain atrophy were thresholded leniently (*p* < 0.01 uncorrected over the whole brain volume) in order to capture any significant grey matter structural changes in relation to functional activation profiles from the fMRI analysis.

#### 2.5.3 Demographic and behavioural data analyses

Demographic data were compared between the healthy control and AD groups using two sample *t*-tests (gender differences were assessed using a Pearson's chi-square test of distribution); neuropsychological data were compared using non-parametric Wilcoxon rank-sum tests. Tone detection thresholds on audiometry screening and performance on post-scan behavioural tasks on experimental stimuli were analysed using linear regression models with clustered, robust standard error due to the model residuals holding non-normal distributions. In the audiometry analysis, the main effect of patient group was assessed while controlling for age and frequency type, as well as assessing for any interaction between group and frequency.

In the analysis of post-scan behavioural data, a ‘cocktail party effect’ measure was generated as the d-prime of name detection in the superimposed and interleaved conditions; the main effect of group and any interactions between test type and group were assessed for all test measures (name detection score/segregation detection score/cocktail party d-prime). In the AD group, correlations between individual post-scan test performance measures and peak effect sizes (beta estimates) for fMRI contrasts of interest were assessed using linear regression: name detection performance was correlated with peak activation in the name identification contrast; segregation detection performance with the segregation contrast; and d-prime with the cocktail party effect contrast.

For all tests, the threshold for statistical significance was *p* < 0.05; Wald tests were used to assess the significance of interaction effects.

## 3 Results

### 3.1 General characteristics of experimental groups

The patient and healthy control groups did not differ significantly in age (*t*_(28)_ = 1.51, *p* = 0.14), gender distribution (χ^2^_(1)_ = 0.62, *p* = 0.43) or years of musical training (*t*(27) = 1.60, *p* = 0.12); the healthy control group had on average significantly more years of education (*t*_(28)_ = 2.08, *p* = 0.048), though participants in both groups overall were relatively highly educated (see Table 1). Tone detection thresholds on audiometry testing revealed that group membership did not have a significant effect on detection time in ms (beta = 3420, CI −673 to 7514, *p* = 0.10). There was a significant interaction between group and frequency [*F*(4,30) = 3.14, *p* = 0.03] driven by the effect of frequency type within group rather than any differences between groups.

### 3.2 Post-scan behavioural data

Group performance data for the post-scan behavioural tests are presented in Table 1. There was a significant main effect of test type (name detection/segregation detection: beta = −2.82, CI −4.24 to −1.41, *p* < 0.001) and a strong trend to a main effect of group (beta = −0.88, CI −1.77 to 0.003, *p* = 0.051). There was a significant interaction between group and test type (*F*_(1,29)_ = 9.29, *p* = 0.005): these results were driven by poorer performance of the AD group than the healthy control group on the auditory segregation detection task (*t* = 3.61, *p* = 0.001). Wald tests also revealed significantly superior performance on name than segregation detection in both healthy individuals (*t* = 4.09, *p* < 0.001) and patients (*t* = 6.11, *p* < 0.001). There was no significant interaction between group and ‘cocktail party’ d-prime (*F*_(1,29)_ = 2.75, *p* = 0.11).

### 3.3 Structural neuroanatomical data

**Fig. S2:**
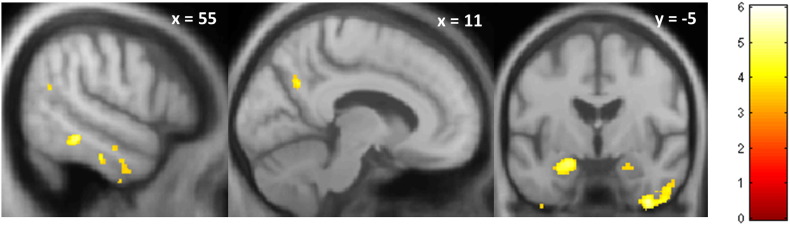
Statistical parametric maps of regional grey matter atrophy in the Alzheimer's disease group compared to the healthy control group based on a voxel-based morphometry analysis of structural brain MR images. Maps are presented on a group mean T1-weighted MR image in MNI space, thresholded leniently at *p* < 0.01 uncorrected for multiple comparisons over whole brain. The colour side bar codes voxel-wise *t*-values of grey matter change. Planes of representative sections are indicated using the corresponding MNI coordinates.

Comparison of the AD and healthy control groups in the VBM analysis revealed the anticipated profile of AD-associated regional grey matter atrophy involving hippocampi, temporal and retrosplenial cortices; statistical parametric maps are presented in Fig. S2 and significant regions of AD-associated grey matter atrophy are summarised in Table S1 in Supplementary Material on-line.

Inline Supplementary Figure S2

Inline Supplementary Table S1

**Table S1:** Regions of significant regional grey matter atrophy in the Alzheimer's disease group compared with the healthy control group in the VBM analysis. Associations shown were significant at threshold *p* < 0.01 uncorrected for multiple comparisons over the whole brain; all significant clusters >50 voxels are shown and peak (local maximum) coordinates are in MNI space. ITG, inferior temporal gyrus; MTG, middle temporal gyrus; PCC, posterior cingulate cortex.

Region	Side	Cluster (voxels)	Peak (mm)			*t*-value
x	y	z
Posterior MTG	R	2187	60	− 36	− 15	6.03
MTG	L	566	− 65	− 21	− 23	4.10
Hippocampus	L	395	− 23	− 4	− 20	4.06
ITG	L	276	− 47	− 33	− 24	4.81
Posterior ITG	L	574	− 57	− 34	− 20	4.70
PCC	R	54	11	− 60	33	4.51
Posterior ITG	L	57	− 48	− 61	− 15	4.15

Inline Supplementary Table S1

### 3.4 Functional neuroanatomical data

**Fig. 2:**
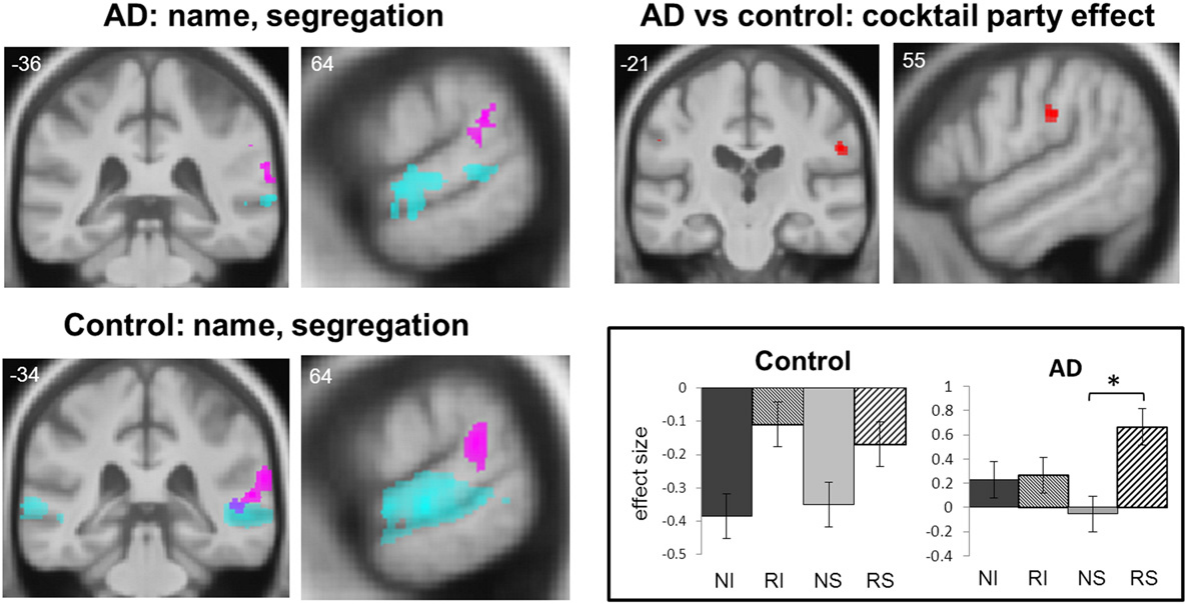
Statistical parametric maps (panels top row, bottom left) of regional brain activation for contrasts of interest in the Alzheimer's disease (AD) and healthy control groups and the between-group ‘cocktail party’ interaction; effect sizes (group mean ±1 standard error peak voxel beta parameter estimates) for each experimental condition at the right supramarginal gyrus peak from the cocktail party contrast are also shown (panel bottom right; * indicates significant difference in effect size between conditions, *p* < 0.01). Statistical parametric maps are rendered on coronal and sagittal sections of the study-specific group mean T1-weighted structural MR image in MNI space; the coordinate of each section plane is indicated and the right hemisphere is shown on the right in all coronal sections. Maps have been thresholded at *p* < 0.001 uncorrected over whole brain for display purposes; activations shown were significant at *p* < 0.05 after family-wise error correction for multiple comparisons over anatomical small volume of interest (see also Table 2). Contrasts were composed as follows: name identification (cyan), [(NS + NI) − (RS + RI)]; auditory object segregation processing (magenta), [(NI + RI) − (NS + RS)]; cocktail party effect (red), [(NI − RI) − (NS − RS)] where NI is own natural name interleaved with babble, NS own natural name superimposed on babble, RI spectrally rotated name interleaved with babble, RS spectrally rotated name superimposed on babble.

Significant neuroanatomical findings from the fMRI analysis are summarised in Table 2; statistical parametric maps and beta parameter estimates for key contrasts and conditions are presented in Fig. 2. All reported contrasts were significant at threshold *p* < 0.05_FWE_, corrected for multiple voxel-wise comparisons within anatomical regions of interest specified by our prior experimental hypotheses. Auditory stimulation (the contrast of all sound conditions versus silence) was associated, as anticipated, with extensive bilateral activation involving the superior temporal gyri in both the AD and healthy control groups; no significant differences between groups were identified and there was no significant activation associated with the ‘reverse’ contrast. Identification of own name compared with spectrally rotated analogues produced extensive bilateral activation of superior temporal gyrus and superior temporal sulcus in both the AD and the healthy control groups; again, no significant differences between groups were identified and there were no significant areas of activation for the ‘reverse’ contrast. In the contrast assessing auditory object segregation processing, right planum temporale and posterior superior temporal gyrus were more activated in the interleaved than superimposed sound conditions (i.e., in the ‘reverse’ contrast: [(NI + RI) − (NS + RS)]) in both the AD and the healthy control groups. Healthy individuals showed additional activation in an inferior parietal junctional area (supramarginal gyrus), however there were no significant differences between participant groups nor any significant activations associated with the ‘forward’ contrast. The contrast to assess the interaction of own name identification with auditory segregation processing (the cocktail party effect) produced no significant activations in the healthy control group but significant activation of right supramarginal gyrus in the AD group. There was a significant difference between groups for this contrast in right supramarginal gyrus.

To further investigate this disease-associated modulation of cocktail party processing in supramarginal gyrus, we conducted an exploratory post hoc analysis of condition effects for both the AD and healthy control groups. Beta parameter estimates in each sound condition relative to the baseline silence condition were compared using pair-wise *t*-tests (Bonferroni corrected, significance threshold *p* < 0.05) at the peak voxel of activation for the cocktail party contrast. In the AD group, activation in the RS condition was significantly greater than both the NS condition (*t*_(12)_ = 3.01, *p* = 0.03) and the RS condition in the healthy control group (*t*_(28)_ = 3.47, *p* = 0.02); there were no other significant sound condition differences within or between groups.

The correlation analysis of peak-voxel beta contrast estimates and post-scan behavioural performance in the AD group revealed no significant relation for name identification (left anterior superior temporal gyrus *r* = −0.23, *p* = 0.45; right anterior superior temporal gyrus *r* = 0.22, *p* = 0.48) but a near-significant trend for segregation processing (right posterior superior temporal gyrus *r* = −0.56, *p* = 0.06). Beta estimates for the cocktail party contrast were significantly correlated with ‘cocktail party’ d-prime (*r* = −0.66, *p* = 0.01).

## 4 Discussion

Here we have shown that the functional neuroanatomy of auditory scene analysis is altered in AD compared to healthy older individuals. This alteration was localised to inferior parietal cortex, a brain region previously implicated as playing a key part both in auditory scene analysis in the healthy brain (Dykstra et al., 2011; Kondo and Kashino, 2009; Kong et al., 2014; Linden et al., 1999) and in the pathogenesis of AD (Seeley et al., 2009; Warren et al., 2012). Our findings build on the growing body of evidence for specific and significant impairments of central auditory function in AD (Gates et al., 1996, 2008, 2011; Golden et al., 2015; Goll et al., 2011, 2012; Golob et al., 2007, 2009; Kurylo et al., 1993; Strouse et al., 1995). The findings show that processes of auditory scene analysis can delineate functional as well as structural neural network alterations in AD based on a relatively naturalistic stimulus that simulates the kind of listening conditions in which these patients commonly report difficulties in daily life. The data further suggest that AD may have a specific computational signature arising from an interaction of cognitive operations that mediate the ‘cocktail party effect’.

The activation profiles of name identification were similar in both the healthy control and AD groups and in accord with previous evidence showing that processing of intelligible speech signals engages distributed superior temporal cortical areas extending beyond auditory cortex (Davis et al., 2011; Meyer et al., 2005; Obleser et al., 2008; Scott et al., 2000). Inclusion of conditions in which name was presented over background babble aligns the present work with previous studies of masked speech processing, which has been shown to engage bihemispheric mechanisms that analyse dynamic spectrotemporal as well as lexical properties of this complex acoustic signal (Scott and McGettigan, 2013). Both patients and healthy individuals were able reliably to discriminate their own names from spectrally rotated versions in post-scan behavioural testing, suggesting that the activation produced by this contrast here indexed name identification per se as well as more generic spectrotemporal template matching and object analysis processes (Billig et al., 2013; Davis et al., 2011; Griffiths and Warren, 2002). It should be noted that the name identification contrast here spanned a change in the spectrotemporal composition of the acoustic background (natural versus spectrally rotated babble) as well as the foreground name sounds: while the use of a spectrally rotated background was intended to reduce spectral ‘pop-out’ of rotated name sounds, future work might dissect the effects of spectral rotation per se from and template-matching processes using alternative speech degradation procedures and different auditory target objects.

Auditory object segregation processing was associated with activation of more posterior superior temporal and inferior parietal cortex in both the healthy control and AD groups: again, this broadly corroborates previous work in the healthy brain (Dykstra et al., 2011; Gutschalk et al., 2007; Hill and Miller, 2010; Kondo and Kashino, 2009; Kong et al., 2014; Linden et al., 1999; Overath et al., 2010; Wilson et al., 2007; Wong et al., 2009). While the direction of this effect here might seem somewhat counter-intuitive (on the basis that segregation of superimposed sounds should require ‘more’ computational processing than resolved interleaved sounds: (Deike et al., 2004, 2010; Gutschalk et al., 2007; Nakai et al., 2005; Wilson et al., 2007), it is consistent with certain previous observations (Hwang et al., 2006; Mustovic et al., 2003; Scott and McGettigan, 2013; Voisin et al., 2006). Speech in noise has been associated with reduced activation of posterior superior temporal cortex compared with clear speech (Hwang et al., 2006): this might reflect reduced intelligibility of the superimposed speech conditions (Scott and McGettigan, 2013) or (more plausibly, in the present case) enhanced engagement of the putative cortical template matching algorithm by intermittent ‘glimpses’ of the salient name sounds (Griffiths and Warren, 2002). Such ‘glimpses’ may have facilitated neural template matching by establishing expectancies over the course of a trial, a process that would be more efficient if name sounds are presented clearly (interleaved) rather than superimposed on background noise. Posterior temporal and temporo-parietal cortex may be particularly sensitive to expectancies of this kind in sound scenes (Mustovic et al., 2003; Voisin et al., 2006). Although this study was not designed to assess lateralised cerebral processing mechanisms explicitly and apparent laterality effects should therefore be interpreted with caution, it is of interest that auditory segregation processing produced peak activation in the right hemisphere in both the healthy control and AD groups here. The correlation with behavioural performance in our AD group further suggested that activity in this region may be required for successful auditory object segregation. Taken together, these findings are consistent with previous evidence that right (non-dominant) temporo-parietal cortex may play a critical role in auditory spatial analysis (Arnott et al., 2004; Krumbholz et al., 2005; Zimmer et al., 2003). This role may be modulated by stimulus characteristics, such as the use of spectrally rotated speech here (Scott et al., 2004).

Arguably more surprising was the lack of significant neuroanatomical differences between the present AD and healthy control groups for the main effect of auditory segregation processing, particularly given that (as anticipated) the AD group showed clearly reduced ability to discriminate superimposed from interleaved sound conditions in the post-scan behavioural test. This may at least in part reflect power to detect effects: functional neuroanatomical differences might emerge with larger patient cohorts. However, stimulus and task factors may also be relevant. In this initial study, we set out to use a paradigm simulating relatively realistic, everyday listening conditions that expose difficulties in patients with AD relative to healthy older people. The use of a babble background is likely to have entailed elements of both energetic and informational masking of superimposed speech sounds (Scott and McGettigan, 2013): it may be that cortical computations associated with disambiguating particular maskers are differentially vulnerable in AD (and of course, in a ‘real’ cocktail party scenario the relative proportion of energetic and informational masking effects is likely to vary unpredictably). Furthermore, it is known that masker level has complex effects on brain activation profiles during auditory scene analysis, particularly in the ageing brain (Scott and McGettigan, 2013; Wong et al., 2009): use of more demanding, reduced signal to noise ratios might amplify any functional neuroanatomical alterations associated with AD. Moreover, as our interest here was in perceptual processing mechanisms that eschew task strategy or difficulty effects, our paradigm did not employ an output task: an active segregation task requirement (as in the post-scan behavioural test here) might well reveal an AD-associated functional anatomical signature.

The interaction of template matching and object segregation in inferior parietal cortex during auditory scene analysis — the cocktail party effect — emerged as the key processing signature differentiating AD from the healthy older brain in this study. This is in line with evidence from previous work that this core computation is particularly vulnerable to cortical network dysfunction in AD (Goll et al., 2012; Warren et al., 2012). The anatomical locus of the effect in supramarginal gyrus further corroborates previous work implicating this area both in auditory scene analysis in the healthy brain and in the network pathophysiology of AD. In the healthy auditory brain, supramarginal gyrus has been linked to auditory target detection, spatial attention and streaming (Dykstra et al., 2011; Kondo and Kashino, 2009; Kong et al., 2014; Linden et al., 1999; Nakai et al., 2005; Scott and McGettigan, 2013), suggesting this region is involved in preparation of orienting and other behavioural responses to the auditory environment (Hickok and Poeppel, 2007; Warren et al., 2005). In AD, dysfunction of temporo-parietal junction is well documented as a hub of the critical, so-called ‘default mode network’ (Buckner et al., 2008; Greicius and Menon, 2004; Raichle et al., 2001; Seeley et al., 2009; Warren et al., 2012). Deconstruction of the complex ‘cocktail party’ interaction here (Fig. 2) revealed that this effect in supramarginal gyrus arose from increased differential activation in the AD group for processing spectrally rotated name versus own natural name sounds superimposed on the acoustic background: activation was enhanced in the AD group compared with healthy controls. Together these profiles suggest that AD may lead to abnormally enhanced activation (or failed deactivation) of inferior parietal cortex during analysis of the incoming sound stream. Dynamic activity shifts in inferior parietal components of the default mode network may normally act to maximise processing efficiency; such shifts might maintain sensitivity to aberrant sensory stimuli that are more difficult to match against stored templates (Chiang et al., 2013; Newman and Twieg, 2001), whereas this sensitivity may be blunted in AD. Modulation of inferior parietal cortex activity could facilitate overall network responsivity to salient auditory and other environmental events, consistent with the proposed ‘sentinel’ function of the default mode network in the healthy brain and its blighting in AD (Buckner et al., 2008; Gilbert et al., 2007).

The present paradigm employed a highly salient, self-referential stimulus (own name): the default mode network including inferior parietal cortex is likely to play a fundamental role in integrating inward representations of self with the world at large, and this process may be disrupted in AD (Molnar-Szakacs and Uddin, 2013). Hearing one's own name may therefore constitute a particularly potent probe of the default mode network and evolving network dysfunction during the development of AD. The key disease interaction here is unlikely to be simply a manifestation of the regional brain atrophy that accompanies AD. With the caveat that structural and functional neuroimaging modalities are generally difficult to compare directly, the location of the functional alteration in supramarginal gyrus lays beyond the zone of significant grey matter atrophy identified in a leniently-thresholded VBM analysis on the same participant groups (see Fig. S2). It is well established that regional brain dysfunction in AD occurs early in the disease course and may lead to structural brain damage (Herholz et al., 2002; Scahill et al., 2002): while it is of course unlikely that inferior parietal cortex in the AD group here was structurally entirely normal, the functional and structural profiles together imply that volume loss alone did not entirely account for the AD-associated functional alteration observed. The direction and selectivity of the functional effect here also speak to this issue: patients with AD showed abnormally enhanced regional cortical activation under particular auditory conditions relative to healthy individuals, rather than simply uniformly attenuated activation as one might anticipate were this wholly dependent on regional grey matter volume. Detection of such aberrant activity increases is an important motivation for employing functional alongside structural neuroimaging techniques in the characterisation of AD and other neurodegenerative diseases (Warren et al., 2012).

The correlation of inferior parietal activity with a behavioural measure of successful cocktail party processing in our AD patients suggests that enhanced activation of this region may help maintain some compensatory function in AD, albeit at the expense of processing inefficiency. However, the present paradigm does not resolve the nature of any relation between activation profiles and behavioural output, since this can only be directly assessed using in-scanner behavioural tasks. The disambiguation of compensatory from aberrantly increased cerebral activity is a key issue in the interpretation of functional neuroimaging changes in neurodegenerative disease (Elman et al., 2014) and a clear priority for future work. Our focus here was to assess AD effects on computational brain mechanisms that might be regarded as obligatory, prior to any modulatory effect from task demands. Ultimately, however, direct assessment of task effects on brain activation profiles will be required both to delineate the network pathophysiology of AD and to evaluate the potential of fMRI as a disease biomarker.

This study has several limitations that suggest directions for further work. Case numbers here were relatively small; in future, it will be important to study larger patient cohorts representing a broader phenotypic spectrum of AD. This is particularly relevant to the delineation of functional profiles that may distinguish typical amnestic AD from major variant syndromes, notably posterior cortical atrophy which is associated with disproportionately prominent impairment of spatial analysis (Warren et al., 2012); and separate AD from other neurodegenerative diseases. Related to this, the AD group here was relatively young: while this will have tended to minimise confounding effects from vascular and other comorbidities, therefore yielding a purer index of functional alterations associated with AD pathology, future work should extend recruitment to include older individuals who represent the major burden of AD in the wider community. Indeed, the brain mechanisms that support auditory scene analysis even in the healthy ageing brain need to be more completely defined. The present auditory paradigm raises unresolved issues that should be investigated in more detail: these include perceptual difficulty effects on the processing of sound conditions within healthy control and patient cohorts; target, masking stimulus, and signal-to-noise effects; and the impact of explicit task requirements. The clinical relevance of functional alterations will ultimately only be established by studying patients at different disease stages and by correlating brain signatures with daily life symptoms, for which more serviceable indices of impaired auditory scene analysis are ideally also required. From a neuroanatomical perspective, in this study we have adopted a directed, region-of-interest approach to assess the neural substrates of auditory scene analysis, informed by the study of the healthy younger brain. Larger cohorts would provide greater power to delineate neuroanatomical correlates beyond these canonical regions, both in the healthy ageing brain and in AD; this may in turn require multi-centre studies to assess the generalisability of findings. In addition, regional functional alterations occur within distributed brain networks and will only be fully defined using connectivity-based techniques, an issue of special pertinence to neurodegenerative diseases underpinned by large-scale neural network disintegration (Seeley et al., 2009; Warren et al., 2012). Acknowledging these various limitations, the present study suggests that auditory scene analysis may constitute a novel and useful paradigm for identifying novel computational signatures of AD and provides a rationale for further systematic investigation with coordinated behavioural and neuroanatomical approaches.

## Figures and Tables

**Table 1:** General demographic, clinical and behavioural data for participant groups.

Characteristics	Healthy controls	AD
*General*		
No (m:f).	17 (8:9)	13 (8:5)
Age (years)	68.3 (3.9)	65.7 (5.6)
Education (years)	15.8 (3.0)	13.4 (3.2)^a^
Musical training (years)	1.7 (2.7)	3.0 (2.7)
MMSE	28.8 (0.9)	19.7 (6.5)^a^
Symptom duration (years)	−	4.9 (1.7)
		
*Neuropsychological assessment*		
*General intellect: IQ*		
WASI verbal IQ	118.6 (8.1)	87.1 (22.3)^a^
WASI performance IQ	118.1 (15.1)	83.5 (17.4)^a^
NART estimated premorbid IQ	119.7 (5.7)	103.9 (16.5)^a^
		
*Episodic memory*		
RMT words (/50)	46.2 (2.8)	**30.6 (6.9)^a^**
RMT faces (/50)	43.1 (4.6)	**33.5 (7.1)^a^**
		
*Executive skills*		
WASI block design (/71)	42.4 (16.6)	12.6 (13.7)^a^
WASI matrices (/32)	29.4 (14.9)	12.8 (9.6)^a^
WMS-R digit span forward (/12)	8.6 (1.8)	6.1 (2.1)^a^
WMS-R digit span backward (/12)	6.6 (2.2)	4.5 (2.8)^a^
D-KEFS Stroop^b^ colour (s)	33.0 (7.1)	**53.3 (18.0)^a^**
D-KEFS Stroop^b^ word (s)	22.4 (4.5)	**41.4 (25.6)^a^**
D-KEFS Stroop^b^ interference (s)	62.2 (16.7)	**102.1 (32.9)^a^**
		
*Verbal skills*		
WASI vocabulary (/80)	68.1 (4.5)	45.2 (20.2)^a^
WASI similarities (/48)	41.1 (9.0)	23.1 (12.8)^a^
GNT (/30)	24.9 (3.2)	**12.9 (8.5)^a^**
BPVS (/150)	146.8 (3.0)	123.8 (28.8)^a^
NART^c^ (/50)	41.2 (4.6)	30.2 (12.2)^a^
		
*Posterior cortical skills*		
GDA^d^ (/24)	15.6 (3.5)	6.4 (4.9)^a^
VOSP object decision (/20)	18.2 (1.5)	14.8 (2.9)^a^
		
*Post-scan behavioural tasks*		
Name detection (/20)	19.9 (0.3)	19.0 (1.5)
Segregation detection (/20)^c^	17.1 (2.7)	12.2 (4.1)^a^

Values are mean (standard deviation, std) unless otherwise stated. Raw data are shown for neuropsychological tests (maximum score in parentheses); bold indicates mean raw score <5th percentile based on published norms.

AD, patient group with typical Alzheimer's disease; BPVS, British Picture Vocabulary Scale (Dunn et al., 1982); D-KEFS, Delis Kaplan Executive System (Delis et al., 2001); GDA, Graded Difficulty Arithmetic (Jackson and Warrington, 1986); GNT, Graded Naming Test (McKenna and Warrington, 1983); L, left; MMSE, Mini-Mental State Examination score; NART, National Adult Reading Test (Nelson, 1982); R, right**;** RMT, Recognition Memory Test (Warrington, 1984); VOSP, Visual Object and Spatial Perception Battery (Warrington and James, 1991); WASI, Wechsler Abbreviated Scale of Intelligence (Wechsler, 1999); WMS-R, Wechsler Memory Scale, Revised (Wechsler, 1987).

a Significantly different to healthy control group.

b Three patients did not complete all sub-sections of this task.

c One patient did not complete this task.

d Four patients were unable to complete this task.

**Table 2:** Summary of fMRI data for experimental contrasts of interest in participant groups.

Group	Contrast	Region	Side	Cluster (voxels)	Peak (mm)			*t*-Value	*p*-Value
x	y	z
Healthy controls	Sound versus silence	HG	L	4344	−44	−21	4	12.10	<0.001
Mid STG	R	4635	60	−12	−2	11.56	<0.001
Name identification^a^	Mid STG/STS	L	1788	−56	−13	−2	10.31	<0.001
R	1989	66	−16	−5	11.38	<0.001
Post STG	L	219	−62	−24	1	8.22	0.001
R	35	65	−18	6	5.46	0.039
Segregation processing^b^	PT/ SMG	R	172	65	−36	19	5.68	0.028
AD patients	Sound versus silence	Mid STG	L	3639	−56	−21	3	23.14	<0.001
Post STG	R	3990	54	−22	10	11.79	<0.001
Name identification	Ant STG/STS	L	652	−59	0	−15	8.34	0.003
R	1073	62	−1	−6	8.34	0.003
Segregation processing	Post STG/PT	R	67	65	−37	24	6.48	0.047
Cocktail party effect^c^	SMG	R	39	55	−22	28	6.47	0.048
Patients > controls	Cocktail party effect	SMG	R	57	55	−21	28	6.06	0.002

Statistical parametric data summarising regional brain activations for contrasts between experimental conditions of interest, in each participant group and between groups. All contrasts shown are thresholded at *p* < 0.05_FWE_ after multiple comparisons correction in pre-specified anatomical small volumes.

NS own natural name superimposed on babble, RI spectrally rotated name interleaved with babble, RS spectrally rotated name superimposed on babble; no significant activations were identified for the ‘forward’ segregation contrast [(NS + RS) − (NI + RI)] in either participant group, for the cocktail party contrast in the healthy control group or for auditory stimulation, name identification or segregation processing between groups. AD, Alzheimer's disease; Ant, anterior; HG, Heschl's gyrus; Post, posterior; PT, planum temporale; SMG, supramarginal gyrus; STG, superior temporal gyrus; STS, superior temporal sulcus.

a Contrast [(NS + NI) − (RS + RI)].

b Contrast [(NI + RI) − (NS + RS)].

c Contrast [(NI − RI) − (NS − RS)] where NI is own natural name interleaved with babble, NS own natural name superimposed on babble, RI spectrally rotated name interleaved with babble, RS spectrally rotated name superimposed on babble.

## Appendix A Supplementary data

Exemplar stimulus: NI, own natural name sound interleaved with babble.

Exemplar stimulus: NS, own natural name sound superimposed on babble.

Exemplar stimulus: RI, spectrally rotated name sound interleaved with (spectrally rotated) babble.

Exemplar stimulus: RS, spectrally rotated name sound superimposed on (spectrally rotated) babble.
