# Supplementary material for: Functional neuroanatomy of auditory scene analysis in Alzheimer's disease
Source: Neuroimage Clin. 2015 Feb 28;7:699–708. doi: 10.1016/j.nicl.2015.02.019 (PMC4446369; doi:10.1016/j.nicl.2015.02.019)
Supplement: Inline Supplementary Table S1 [file mmc1.docx]

| **Region** | **Side** | **Cluster**  (voxels) | **Peak** (mm) | | | ***t*-value** |
| --- | --- | --- | --- | --- | --- | --- |
|  |  |  | x | y | z |  |
| Posterior MTG | R | 2187 | 60 | -36 | -15 | 6.03 |
| MTG | L | 566 | -65 | -21 | -23 | 4.10 |
| Hippocampus | L | 395 | -23 | -4 | -20 | 4.06 |
| ITG | L | 276 | -47 | -33 | -24 | 4.81 |
| Posterior ITG | L | 574 | -57 | -34 | -20 | 4.70 |
| PCC | R | 54 | 11 | -60 | 33 | 4.51 |
| Posterior ITG | L | 57 | -48 | -61 | -15 | 4.15 |

**Table S1**. Regions of significant regional grey matter atrophy in the Alzheimer’s disease group compared with the healthy control group in the VBM analysis. Associations shown were significant at threshold p<0.01 uncorrected for multiple comparisons over the whole brain; all significant clusters >50 voxels are shown and peak (local maximum) coordinates are in MNI space. ITG, inferior temporal gyrus; MTG, middle temporal gyrus; PCC, posterior cingulate cortex.
